# IL-15 is decreased upon CsA and FK506 treatment of acute rejection following heart transplantation in mice

**DOI:** 10.3892/mmr.2014.2703

**Published:** 2014-10-20

**Authors:** ZHIYONG YU, XIAOPING ZHOU, SONGFENG YU, HAIYANG XIE, SHUSEN ZHENG

**Affiliations:** 1 Department of Surgery, Division of Hepatobiliary and Pancreatic Surgery, First Affiliated Hospital, School of Medicine, Zhejiang University, Hangzhou, Zhejiang 310003, P.R. China; 2 Key Laboratory of Combined Multi-organ Transplantation, Ministry of Public Health, Hangzhou, Zhejiang 310003, P.R. China; 3 Key Laboratory of Organ Transplantation, First Affiliated Hospital, School of Medicine, Zhejiang University, Hangzhou, Zhejiang 310003, P.R. China

**Keywords:** rejection, interleukin-15, heart transplantation, mice, cyclosporine A, FK506

## Abstract

The aim of this study was to investigate the effect of cyclosporine A (CsA) and tacrolimus (FK506) on interleukin-15 (IL-15) production during acute rejection following heart transplantation in mice. Inbred male Balb/c (H-2d) and C57BL/6 (H-2b) mice were used to establish a heterotopic intra-abdominal cardiac transplantation model. The mice were divided in four groups: syngeneic control, allogeneic acute rejection, allogeneic rejection treated with CsA, and allogeneic rejection treated with FK506. The expression of IL-15, IL-2, and tumor necrosis factor-α (TNF-α) was measured using reverse transcription-polymerase chain reaction (RT-PCR) and western blotting. A low level of IL-15 was detected in transplanted hearts of the control group, with a significant increase observed in the allogeneic acute rejection group. Compared to the allogeneic acute rejection group, IL-15 expression was significantly decreased in the CsA-and FK506-treated allogeneic rejection groups. The TNF-α expression pattern was similar to that of IL-15 in all groups. IL-2 expression was increased in the allogeneic acute rejection group and was inhibited in mice treated with CsA and FK506. In conclusion, increased IL-15 expression in rejected murine heart grafts may be reduced by CsA and FK506 *in vivo*.

## Introduction

Organ transplantation is considered the best option for treating patients who suffer from organ failure or dysfunction. However, the long-term allograft survival rate remains unsatisfactory due to alloimmune rejection reactions. Thus, treating acute rejection is the only option to improve the long-term allograft survival rate. Acute allograft rejection is thought to be predominantly affected by T-cell mediated processes.

Interleukin-2 (IL-2) is considered to be the unique growth factor for T cells; however, rejection episodes are not always mediated by IL-2. Allograft rejection still occurs despite IL-2/IL-2R blockade (1), knockout of the *IL-2* gene, and *IL-2*/*IL-4* double knockout (2,3). This suggests that other cytokines may be involved in acute IL-2-negative anti-donor responses, such as IL-4, IL-7, IL-9 and IL-15, which can bind to the γ-chain of the IL-2 receptor (IL-2R) complex (4,5).

IL-15 and IL-2 have similar structures. They share several biological activities, including stimulation of the proliferation and differentiation of T cells, and promotion of T cell chemotaxis (6–8). However, unlike IL-2, IL-15 is derived from a wide range of cell types, including activated macrophages, activated vascular endothelial cells, fibroblasts, muscle cells, and epithelial cells (9). Moreover, although IL-15 and IL-2 share the same IL-2Rβ and γ-chain receptor subunits, IL-15 has a unique α-chain, IL-15Rα (6–8). The role of IL-15 in allograft rejection is not clearly defined, although important roles for this cytokine have been suggested in other immunopathologies, such as infectious diseases (10), atherosclerosis (11), human immunodeficiency virus infection (12), and cancer (13–15). A number of studies have shown an increase in the expression of intragraft *IL-15* mRNA in both IL-2-dependent and -independent allograft rejection cases, and suggested that increased IL-15 expression correlates with acute rejection (2,3,16,17), particularly in the case of IL-2-independent rejection. These findings support the hypothesis that IL-15 may substitute IL-2 in the mechanism underlying IL-2-independent rejection.

Cyclosporine A (CsA) and tacrolimus (FK506), are two conventional immunosuppressant drugs widely used in the clinical setting, which are effective in not only preventing acute rejection, but also prolonging allograft survival time by inhibiting IL-2 production. However, the effect of CsA and FK506 on IL-15 production remains unclear. Although some *in vitro* studies have been performed, the results were inconsistent and contradictory (18–20). To date, no study exists that has examined the effects of CsA and FK506 on IL-15 expression *in vivo*. This study evaluated the effects of treatment with CsA and FK506 on the alloimmune responses following heart transplantation in mice, and specifically showed that IL-15 expression is inhibited by both drugs.

## Materials and methods

### Drugs

Stock solutions of CsA and FK506 were prepared as follows: CsA was purchased from Novartis Pharmaceuticals (Basel, Switzerland), and was dissolved in physiological saline to obtain a working concentration of 0.4 mg/ml. The FK506 stock (Fujisawa Pharmaceutical, Osaka Japan) was suspended in phosphate-buffered saline (PBS) to obtain a working concentration of 0.08 mg/ml.

### Animals and groups

C57BL/6 (H-2b) and Balb/c (H-2d) mice, 6–8 weeks old, weighing 18–20 g, were purchased from the Shanghai Laboratory Animal Center of the Chinese Academy of Sciences, and were housed in cages inside a room with a light/dark cycle. Heterotopic intra-abdominal cardiac transplantation was performed with the method reported by (21), with some modifications. Briefly, the hearts of the C57 mice were transplanted into the abdominal cavities of the Balb/c mice, the aortic ascent artery of the C57 mice was connected to the abdomnal aortic artery of the Balb/c mice, and the main pulmonary artery of the C57 mice was connected to the inferior cavae vein of the Balb/c mice. Balb/c (H-2d) mice that received the transplant were randomly divided in four groups: syngeneic control group, where donors were Balb/c mice; allogeneic acute rejection group, where donors were C57BL/6 mice; allogeneic CsA treatment group, where donors were C57BL/6 mice and treatment with CsA was performed by intraperitoneal injection of 10 mg/kg/day CsA from the day of operation [post-operative day (POD) 0] to POD 13; and allogeneic FK506 treatment group, where donors were C57BL/6 mice and recipients were treated with FK506 1.0 mg/kg/day by intraperitoneal injection from POD 0 to POD 13. Each of these four groups was subdivided into four groups (n=5) corresponding to PODs 1, 3, 5 and 7 for sample harvesting, and additional subgroups (n=6) for general observations and measurement of graft survival time. The graft survival time was assessed daily by palpation of the heart graft. All experiments were approved by the Animal Welfare Committee and were performed according to the Laboratory Animal Management Guidelines of the Zhejiang University.

### RNA extraction and reverse transcription (RT)

For RT-polymerase chain reaction (PCR) analysis, specimens were snap frozen in liquid nitrogen and stored at −80°C. Total RNA was extracted with the Gibco^®^ TRIzol reagent according to the manufacturer’s instructions (Thermo Fisher Scientific, Waltham, MA, USA). First strand cDNA synthesis was performed as follows: 4 μg isolated RNA and 3 μl random primers (Fermentas, Thermo Fisher Scientific, Waltham, MA, USA) were mixed with double distilled (dd) H_2_O, in order to obtain a final volume of 11 μl. The reaction mixture was incubated at 70°C for 5 min, and at 0°C for 5 min. A total of 5 μl 5× MMLV-RT reaction buffer, 2 μl 10 mM dNTP, 1 μl ddH_2_0 and 1 μl MMLV Reverse Transcriptase (Fermentas) was subsequently added and the samples were incubated at 42°C for 60 min, and at 70°C for 10 min to terminate the reaction. The synthesized cDNA was stored at −20°C.

### PCR analysis

PCR analysis was conducted as follows: synthesized cDNA (2 μl) was amplified in a 25 μl reaction volume containing sense and anti-sense primers of each cytokine (Table 1). The reagents were used first to rule out failure of the reverse transcriptase reaction and PCR amplification, and second to detect gross variation in cDNA quantity among the samples. The samples were amplified in a PTC-200 Peltier Thermal cycler (MJ Research, Inc., Watertown, MA, USA). The conditions were optimized for each primer pair to avoid the amplification of non-specific products (Table I).

### Semi-quantitative mRNA analysis

Amplified products and the Fermentas^®^ pUC19 DNA/*Msp*I (*HpA*II) marker 23 (Thermo Fisher Scientific) were analyzed by electrophoresis on 1.5% agarose gels containing ethidium bromide. Images of the gels were acquired and the band intensity was analyzed using the Kodak analysis of the gel image software (Life Technologies, Grand Island, NY, USA). To correct for variations in the mRNA concentration in each sample, the densitometry value for each cytokine was divided by the corresponding value of β-actin.

### Protein extraction, titration and storage

The heart grafts were lysed in ice-cold tissue lysis buffer, containing 0.25% NP-40, 125 mM KCl, 10 mM MgCl_2_, 60 mM HEPES (pH 7.9), 0.5 mM DTT, 0.5mM phenylmethylsulfonyl fluoride, 10 μg/l aprotinin and 10 μg/l leupeptin (all Sangon Biotech Co., Ltd., Shanghai, China). The cell debris was removed by centrifugation and the protein-containing supernatant was removed and titrated according to the specifications of the DC™ Protein Assay kit (Bio-Rad Laboratories, Inc., Hercules, CA, USA), and stored at −80°C.

### Western blotting

The proteins were subjected to 15% sodium dodecyl sulfate-polyacrylamide gel electrophoresis, and transferred to nitrocellulose membranes (EMD Millipore, Billerica, MA, USA). The membranes were blocked with 5% nonfat dry milk in PBS and incubated with biotinylated goat anti-mouse IL-15 antibody (1:1,000; R&D Systems, Minneapolis, MN, USA) or mouse anti-mouse tumor necrosis factor-α (TNF-α) antibody (1:1,000; Perbio Science AB, Helsingborg, Sweden) at 4°C overnight. After washing with PBS, the membranes were incubated with horseradish peroxidase (HRP)-conjugated anti-goat or anti-mouse immunoglobulin (Ig)G (both at 1:1,500; Dako, Glostrup, Denmark). The bands were visualized using the Enhanced Chemiluminescence Blotting system (Santa Cruz Biotechnology, Inc., Dallas, TX, USA). The blots were developed on X-ray film (Eastman Kodak, Rochester, NY, USA).

### Histologic evaluation

Heart grafts were excised, covered with formalin, embedded in paraffin wax, sectioned and stained with hematoxylin and eosin. They were then observed under a microscope (Leica DM3000; Leica Microsystems, Wetzlar, Germany).

### Statistical analysis

Results were expressed as the mean ± standard deviation (SD). Statistical analysis was performed with the SPSS software (SPSS Inc., Chicago, IL, USA). Independent-sample t-tests and one-way analysis of variance (ANOVA) were used for comparisons between groups of parametric data. A p-value (P) of <0.05 was considered to indicate a statistically significant difference.

## Results

### Heart graft survival time

The heart graft survival times were all longer than 100 days in the control group. In the allogeneic acute rejection group, the heart grafts stopped working by POD 7–9 (8±0.9 days). The mean heart graft survival times of the CsA and FK506 treatment groups were 20.2±4.4 and 17.3±2.1 days, respectively (Table II), which are significantly higher compared to the allogeneic acute rejection group (P<0.001). However, the heart graft survival time did not differ significantly between the CSA and FK506 treatment groups (P=0.1).

### Histology

**Figure 1:**
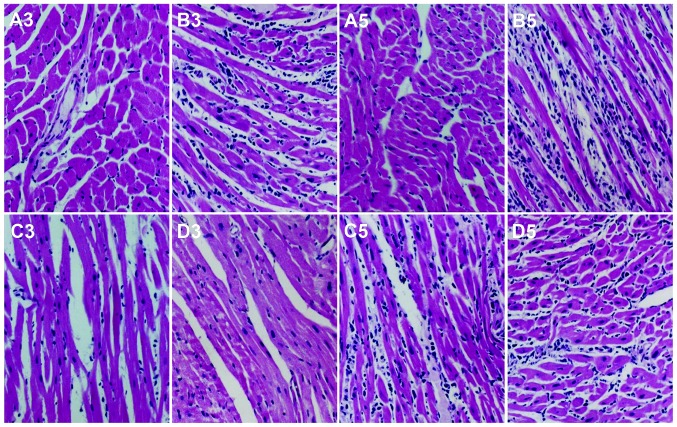
Histological examination of the murine heart grafts, hematoxylin and eosin staining (original magnification, ×200). A, syngeneic control group; B, allogeneic acute rejection group; C, allogeneic cyclosporine A (CsA) treatment group; D, allogeneic tacrolimus (FK506) treatment group. The numbers 3 and 5 in each group refer to the post-operative day (POD) in which the samples were harvested. On POD 3, the heart grafts have a normal histological appearance and show no sign of rejection in the syngeneic control, CsA treatment, and FK506 treatment groups. However, some lymphocyte infiltration and rejection occurs in the heart grafts of the allogeneic acute rejection group. On POD 5, the heart grafts have a normal histological appearance in the syngeneic control group, and rejection occurs in the other groups. Compared to the allogeneic acute rejection group, the degree of lymphocyte infiltration is markedly reduced in the CsA and FK506 treatment groups.

No signs of rejection were detected at any time-point in the control group. Myocardial lymphocyte infiltration was observed in the heart grafts from PODs 3 to 7 in the allogeneic acute rejection group. Rejection was markedly suppressed in the transplanted hearts of the CsA and FK506 treatment groups, with marked mononuclear infiltration observed (Fig. 1).

### Cytokine mRNA expression

**Figure 2:**
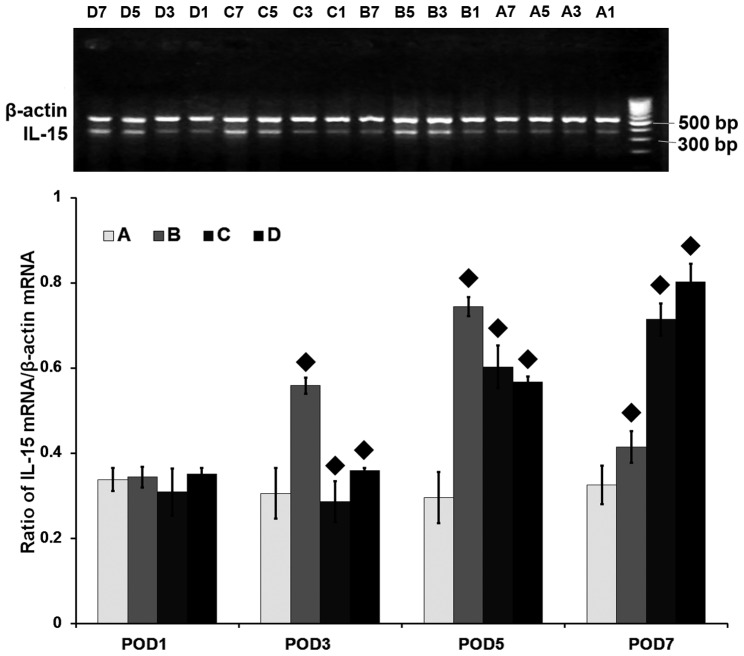
mRNA level of the interleukin-15 gene (*IL-15*) assessed by reverse transcription-polymerase chain reaction in the heart grafts. A, syngeneic control group; B, allogeneic acute rejection group; C, allogeneic cyclosporine A (CsA) treatment group; D, allogeneic tacrolimus (FK506) treatment group. The numbers 1, 3, 5 and 7 in each group refer to the post-operative day (POD) in which the samples were harvested. ♦ indicates statistical significance (P<0.001) on pairwise comparison assessed by a t-test. The *IL-15* gene is expressed at low levels in the syngeneic control group at all time-points or on POD 1 in the other groups. The *IL-15* mRNA level is significantly increased in the allogeneic acute rejection group on PODs 3, 5 and 7 (all, P<0.05), with its highest level reached on POD 5. Compared to the allogeneic acute rejection group, the *IL-15* expression in the CsA and FK506 treatment groups is significantly decreased on PODs 3 and 5 (all, P<0.001), and is again increased on POD 7 (both, P<0.001).

**Figure 3:**
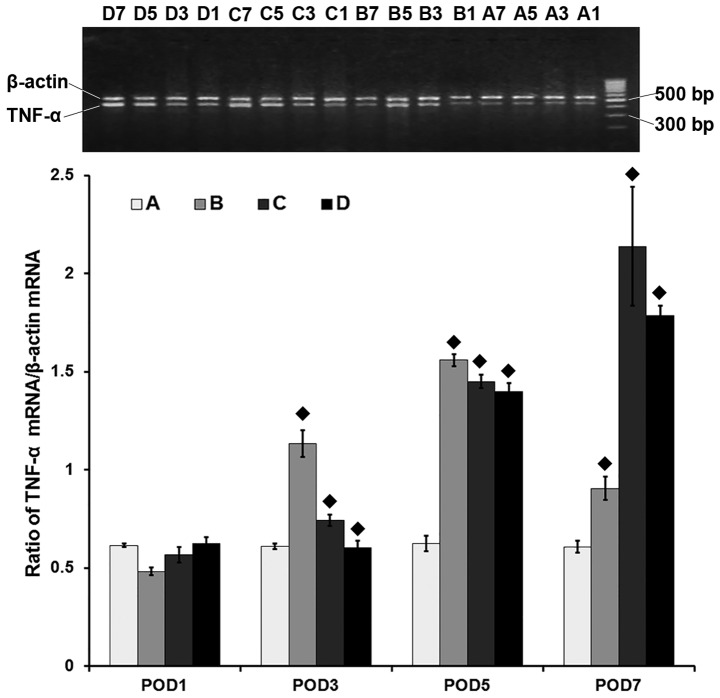
mRNA level of the tumor necrosis factor-α gene (*TNF-α*) assessed by reverse transcription-polymerase chain reaction in the heart grafts. A, syngeneic control group; B, allogeneic acute rejection group; C, allogeneic cyclosporine A (CsA) treatment group; D, allogeneic tacrolimus (FK506) treatment group. The numbers 1, 3, 5 and 7 in each group refer to the post-operative day (POD) in which the samples were harvested. ♦ indicates statistical significance (P<0.001) on pairwise comparison assessed by a t-test. The *TNF-α* gene is expressed at low levels in the syngeneic control group at all time-points or at POD 1 in the other groups. The *TNF-α* mRNA level is significantly increased in the allogeneic acute rejection group on PODs 3, 5 and 7 (all, P<0.001), with its highest level reached on POD 5. Compared to the allogeneic acute rejection group, *TNF-α* expression in the CsA and FK506 treatment groups is significantly decreased on PODs 3 and 5 (all, P<0.001), and is again increased on POD 7 (both, P<0.001).

**Figure 4:**
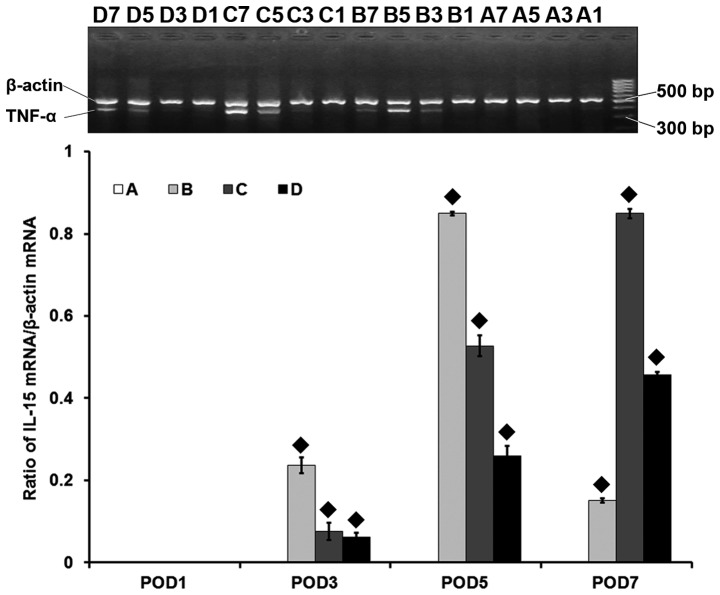
mRNA level of the interleukin-2 gene (*IL-2*) assessed by reverse transcription-polymerase chain reaction in the heart grafts. A, syngeneic control group; B, allogeneic acute rejection group; C, allogeneic cyclosporine A (CsA) treatment group; D, allogeneic tacrolimus (FK506) treatment group. The numbers 1, 3, 5 and 7 in each group refer to the post-operative day (POD) in which the samples were harvested. ♦ indicates statistical significance (P<0.001) on pairwise comparison assesssed by a t-test. The *IL-2* mRNA is not detected in the syngeneic control group at any time point or on POD 1 in the other groups. Its level is significantly increased in the allogeneic acute rejection group on PODs 3, 5 and 7 (all, P<0.001,) reaching its highest level on POD 5. Compared to the allogeneic acute rejection group, *IL-2* expression in the CsA and FK506 treatment groups is significantly decreased on PODs 3 and 5 (all, P<0.001), and is again increased on POD 7 (both, P<0.001).

The *IL-15* mRNA was detected at low levels in the transplanted hearts of the control group at all postoperative time-points. Compared to the control group, the *IL-15* expression was significantly increased in the allogeneic acute rejection group on PODs 3, 5 and 7 (P=0.02, P<0.001 and P=0.03, respectively). The expression of *IL-15* mRNA peaked on POD 5 in the allogeneic acute rejection group. In the CsA and FK506 treatment groups, the *IL-15* mRNA was detected at low levels on PODs 1 and 3, but its level increased from PODs 5 to 7. Compared to the allogeneic acute rejection group, the expression of *IL-15* was significantly inhibited on PODs 3 and 5 in the CsA and FK506 treatment groups (Fig. 2). The expression pattern of the *TNF-α* mRNA was similar to that of *IL-15* in all groups (Fig. 3). The *IL-2* mRNA level was undetectable in the control group and significantly increased in the allogeneic acute rejection group on PODs 3, 5 and 7 (all, P<0.001), with a peak on POD 5. In the CsA and FK506 treatment groups, the *IL-2* mRNA could not be detected on PODs 1 and 3, but ts level increased from PODs 5 to 7. Compared to the allogeneic acute rejection group, the *IL-2* expression was significantly inhibited on PODs 3 and 5 in the CsA and FK506 treatment groups (Fig. 4).

### Cytokine protein expression

**Figure 5:**
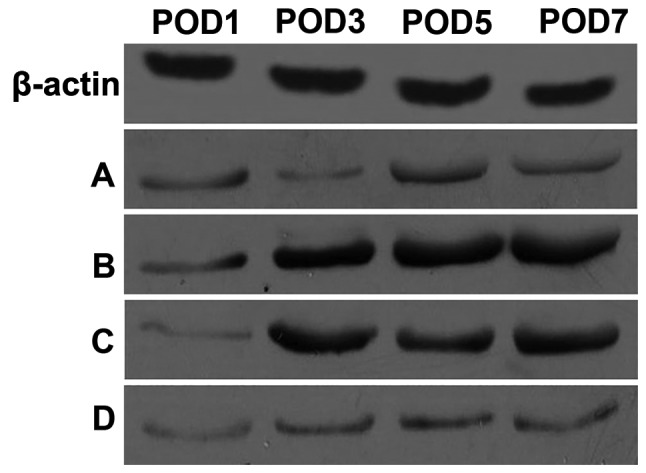
Interleukin-15 (IL-15) western blot. A, syngeneic control group; B, allogeneic acute rejection group; C, allogeneic cyclosporine A (CsA) treatment group; D, allogeneic tacrolimus (FK506) treatment group. The IL-15 protein is expressed at low levels in the syngeneic control group at all time-points. Compared to the syngeneic control group, IL-15 protein expression is increased from postoperative day (POD) 3 and until POD 7 in the allogeneic acute rejection group. In the CsA and FK506 treatment groups, IL-15 protein expression is decreased at all time-points compared to the allogeneic acute rejection group.

**Figure 6:**
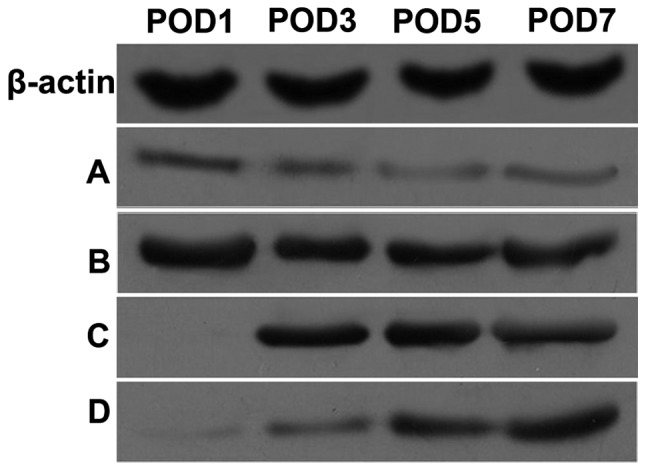
Tumor necrosis factor-α (TNF-α western blot. A, syngeneic control group; B, allogeneic acute rejection group; C, allogeneic cyclosporine A (CsA) treatment group; D, allogeneic tacrolimus (FK506) treatment group. The TNF-α protein is expressed at low levels in the syngeneic control group at all time-points. Compared to the syngeneic control group, TNF-α protein expression is increased from postoperative day (POD) 1 and until POD 7 in the allogeneic acute rejection group. In the CsA and FK506 treatment groups, TNF-α protein expression is decreased at all time-points compared to the allogeneic acute rejection group.

Low levels of the IL-15 and TNF-α proteins were detected in the heart grafts of the control group at all time-points. Compared to the control group, IL-15 protein expression was significantly increased from PODs 3 to 7, and TNF-α protein expression was increased from PODs 1 to 7 in the heart grafts of the allogeneic acute rejection group. In the CsA and FK506 treatment groups, despite the gradual and slight increase from PODs 3 to 7, the expression of IL-15 (Fig. 5) and TNF-α (Fig. 6) was reduced at all time-points relative to the allogeneic acute rejection group.

## Discussion

IL-15, a T cell growth factor, is derived from non-lymphocytes, such as activated macrophages, activated vascular endothelial cells, fibroblasts, and muscle cells. It shares a number of common biological activities with IL-2, including stimulation of T cell, B cell, and natural killer cell proliferation, as well as a T cell chemoattractant activity (6–8). Recent studies showed that IL-15 expression is increased in certain types of immune-mediated tissue injury, such as allograft rejection (4,5,10–12,15). There is considerable interest in the potential role of this cytokine in the pathogenesis of tissue destruction, particularly in the context of alloimmune reactions.

In the present study, the level of the *IL-15* mRNA was low but still detectable in non-rejected heart grafts in the syngeneic control group, whereas it was significantly increased in rejected heart grafts in the acute rejection group. The low level of *IL-15* mRNA expressed may be attributed to cardiac muscle cells, which produce low levels of IL-15 (22). The increased level of *IL-15* mRNA is likely primarily derived from the infiltrating activated monocytes/macrophages in the rejected heart graft, since these cells produce high levels of IL-15 (22). Numerous monocyte/macrophage infiltrates are observed in acutely rejected allografts (23,24). Unlike *IL-15*, the expression of *IL-2* was not detected in non-rejected heart grafts and was significantly increased in rejected heart grafts. The difference between the expression patterns of *IL-15* and *IL-2* may be due to the origin of these two cytokines.

Increased *IL-15* expression appeared associated with the presence of acute rejection in the present study. The strongest expression of the *IL-15* gene was observed on POD 5, the same day at which lymphocyte infiltration peaked in the rejected heart grafts according to the pathological examination.

It is well known that both CsA and FK506 can inhibit IL-2 expression and prolong allograft survival time by blocking calcineurin (25,26). However, the relationship between IL-15 expression and the administration of CsA or FK506 is rarely reported, and only three *in vitro* studies have been performed (18–20); two of these (19,20) reported that the production of IL-15 is unaffected by CsA, but one (18) reported that CsA administration decreases the level of IL-15 in a dose-dependent manner. The contradictory results between these studies may be due to the different cell lines used. In the present study, we demonstrated that IL-15 expression, at both the mRNA and the protein level, is reduced by CsA or FK506 treatment (Figs. 2 and 5). However, we observed a discordance between IL-15 mRNA and protein expression. In the allogeneic acute rejection group, the *IL-15* mRNA level was increased on POD 3, peaked on POD 5, and decreased on POD 7, although the expression of IL-15 was not completely blocked. Notably, on POD 7, the *IL-15* expression in the heart grafts treated with CsA and FK506 increased again. This finding may be related to the dose of the immunosuppressants used in this study. It is likely that CsA or FK506 induce a decrease in the level of *IL-15* in a dose-dependent manner *in vivo,* and increased doses of CsA or FK506 may result in complete inhibition of *IL-15*. However, the mechanism by which CsA or FK506 inhibit the expression of *IL-15* remains unclear. In addition, the present study showed that the *TNF-α* mRNA derived from activated monocytes/macrophages is significantly reduced upon administration of CsA and FK506. This finding indicates that the activity of macrophages may be suppressed in these conditions. We conclude that CsA and FK506 may affect the production of IL-15 via interactions with the macrophages.

In conclusion, IL-15, a non-T cell-derived cytokine, is involved in acute rejection following heart transplantation and is partially downregulated *in vivo* in mice by CsA and FK506 treatment. Whether increased doses of CsA or FK506 may result in the complete inhibition of IL-15 production requires further study, while it is also necessary to clarify the relationship between CsA or FK506 treatment and IL-15 expression in clinical studies.

## Figures and Tables

**Table I:** Oligonucleotide sequences for the amplification of mouse cytokine genes.

Cytokine	Product size (bp)	Primer sequences	PCR cycling conditions
IL-2	408	Sense: 5′-AGC TCC ACT TCA AGC TCT AC-3′ Antisense: 5′-GAC AGA AGG CTA TCC ATC TC-3′	94°C, 4 min→94°C, 30 sec, 64°C, 30 sec, 72°C, 30 sec (33 cycles)→72°C, 10 min
IFN-γ	300	Sense: 5′-TGG GGA CTG AAG TCC TAG AAG-3′ Antisense: 5′-TTA CCC AGT CAG GGT TAC TGC TGC TGT G-3′	94°C, 4 min→94°C, 60 sec, 57°C, 60 sec, 72°C, 60 sec (26 cycles)→72°C, 10 min
IL-15	345	Sense: 5′-TCC ATC TCG TGC TAC TTG TG-3′ Antisense: 5′-CAT TCC TTG CAG CCA GAT TC-3′	94°C, 4 min→94°C, 30 sec, 66°C, 30 sec, 72°C, 30 sec (29 cycles)→72°C, 10 min
TNF-α	446	Sense: 5′-AGC CCA CGT AGC AAA CCA CCA A-3′ Antisense: 5′-ACA CCC ATT CCC TTC ACA GAG CAA T-3′	94°C, 4 min→94°C, 30 sec, 64°C, 30 sec, 72°C, 30 sec (33 cycles)→72°C, 10 min
β-actin	539	Sense: 5′-GTG GGC CGC CCT AGG CAC CAA-3′ Antisense: 5′-CTC TTT GAT GTC ACG CAC GAT TTC-3′	94°C, 4 min→94°C, 30 sec, 62°C, 30 sec, 72°C, 30 sec (28 cycles)→72°C, 10 min

PCR, polymerase chain reaction; IL-2, interleukin-2; IFN-γ, interferon-γ; TNF-α, tumor necrosis factor-α; bp, base pair.

**Table II:** The survival time of heart grafts in the different groups.

Group	n	Survival time^a^ (n^b^)
A	8	100 (8)
B	7	7 (2), 8 (3), 9 (2)
C	6	15, 16, 18, 23, 24, 25
D	6	15, 17, 18, 19, 20, 15

a time expressed in days;

b n=1 unless stated otherwise.

A, syngeneic control group; B, allogeneic acute rejection group; C, allogeneic cyclosporine A treatment group; D, allogeneic tacrolimus (FK506) treatment group.
